# Diagnostic accuracy of the Ottawa ankle rule to exclude fractures in acute ankle injuries in adults: a systematic review and meta-analysis

**DOI:** 10.1186/s12891-022-05831-7

**Published:** 2022-09-23

**Authors:** Yolanda E. Gomes, Minh Chau, Helen A. Banwell, Ryan S. Causby

**Affiliations:** 1 Allied Health and Human Performance Unit, University of South Australia, Adelaide, South Australia Australia; 2 South Australia Medical Imaging, Flinders Medical Centre, South Australia, Australia; 3 Faculty of Health, University of Canberra, Australian Capital Territory, Australia

**Keywords:** Ankle, Ankle injuries, Ankle fractures, Sensitivity and specificity, Ankle radiography

## Abstract

**Background:**

Ankle traumas are common presenting injuries to emergency departments in Australia and worldwide. The Ottawa Ankle Rules (OAR) are a clinical decision tool to exclude ankle fractures, thereby precluding the need for radiographic imaging in patients with acute ankle injury. Previous studies support the OAR as an accurate means of excluding ankle and midfoot fractures, but have included a paediatric population, report both the ankle and mid-foot, or are greater than 5 years old. This systematic review and meta-analysis aimed to update and assess the existing evidence of the diagnostic accuracy of the Ottawa Ankle Rule (OAR) acute ankle injuries in adults.

**Methods:**

A systematic search and screen of was performed for relevant articles dated 1992 to 2020. Prospective and retrospective studies documenting OAR outcomes by physicians to assess ankle injuries were included. Critical appraisal of included studies was assessed using the Quality Assessment of Diagnostic Accuracy Studies 2 (QUADAS-2) tool. Outcomes related to psychometric data were pooled using random effects or fixed effects modelling to calculate diagnostic performance of the OAR. Between-study heterogeneity was assessed using the Higgins I2 test, with Spearman’s correlation test for threshold effect.

**Results:**

From 254 unique studies identified in the screening process, 15 were included, involving 8560 patients from 13 countries. Sensitivity, specificity, negative likelihood ratio, positive likelihood ratio and diagnostic odds ratio were 0.91 (95% CI, 0.89 to 0.92), 0.25 (95% CI, 0.24 to 0.26), 1.47 (95% CI, 1.11 to 1.93), 0.15 (95% CI, 0.72 to 0.29) and 10.95 (95% CI, 5.14 to 23.35) respectively, with high between-study heterogeneity observed (sensitivity: I2 = 94.3%, *p* < 0.01; specificity: I2 = 99.2%, *p* < 0.01). Most studies presented with low risk of bias and concern regarding applicability following assessment against QUADAS-2 criteria.

**Conclusions:**

Application of the OAR is highly sensitive and can correctly predict the likelihood of ankle fractures when present, however, lower specificity rates increase the likelihood of false positives. Overall, the use of the OAR tool is supported as a cost-effective method of reducing unnecessary radiographic referral, that should improve efficiency, lower medical costs and reduce waiting times.

**Supplementary Information:**

The online version contains supplementary material available at 10.1186/s12891-022-05831-7.

## Background

Ankle traumas are one of the most common presenting injuries to emergency departments in Australia and worldwide [1, 2]. Ankle trauma can result from fractures, tendon injuries, ligament sprains or tears, each requiring different management plans. Accurate diagnosis and effective management of such injuries is therefore critical. Current emergency practice relies heavily on the use of radiographic assessment for the management of ankle trauma, despite evidence suggesting that they are not always necessary, especially in the case of soft tissue injuries [2, 3]. The Ottawa Ankle Rules (OAR) are a clinical decision tool developed by Stiell et al. [4] to exclude ankle fractures, thereby precluding the need for radiographic imaging in patients with acute ankle injury. These standardised criteria allow clinicians to be more selective in their use of radiographic imaging and minimises unnecessary exposure to ionising radiation [4, 5].

**Fig. 1:**
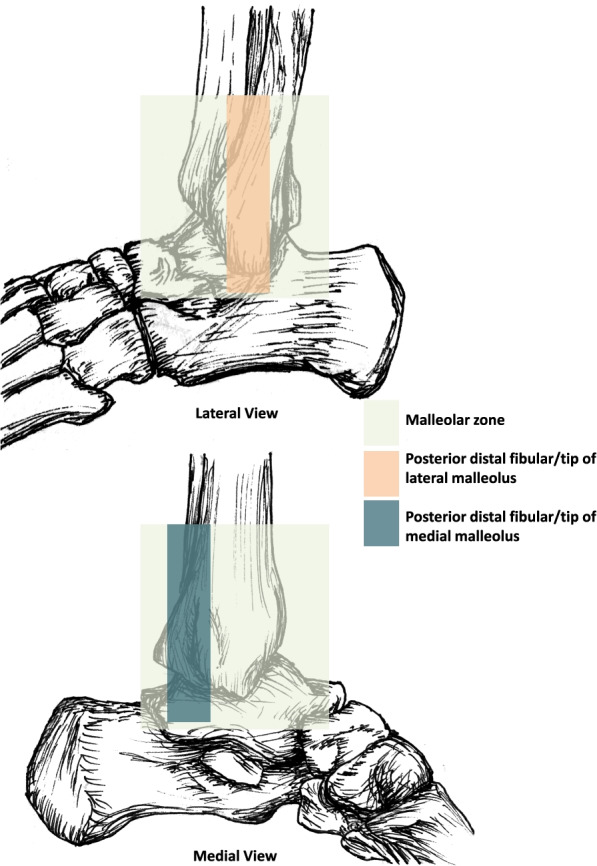
Ottawa Ankle Rules [6] [Image created for and published in PeerJ by the authors under a Creative Commons Attribution License https://doi.org/10.7717/peerj.10152/fig-1]

The OAR (see Fig. 1) state that ankle X-rays are warranted if the patient meets one of the following criteria:

- Pain or bone tenderness in the posterior distal tibia or tip of medial malleolus
- Pain or bone tenderness in the posterior distal fibula or tip of lateral malleolus
- Unable to weight bear immediately after the injury or for four steps in the emergency department

Studies of the OAR in numerous settings have demonstrated high sensitivity and specificity. Specifically, Bachmann et al. conducted a systematic review of studies in which the OAR was used to diagnose ankle fractures [7]. Pooled analysis showed a sensitivity of 97.3% and specificity of 36.6% in the adult population. While the diagnostic accuracy of the test was deemed to be high, no significant change in clinical behaviour was noted, with immediate access to radiography, fear of litigation and lack of dissemination of the rule in primary care identified as potential contributing factors [7]. Furthermore, a recent systematic review and meta-analysis was conducted by Beckenkamp et al. which assessed the diagnostic accuracy of both the Ottawa ankle and midfoot rules and reported on a number of variables including patient age, profession of the assessor and setting [5]. Results for the adult population showed that the rules had a high sensitivity (99.4%) and low negative likelihood ratio (0.02%), and the meta-analysis demonstrated that the profession of the assessing clinician did not affect the accuracy of the rules [5]. Notably, the sensitivity of the OAR was significantly higher in adults than in children [5].

Whilst previous studies support the OAR as an accurate means of excluding ankle and midfoot fractures, they have included a paediatric population, often report both the ankle and mid-foot, and/or are equal or greater than 5 years old. The primary objective of this study is to conduct a review of current literature to determine whether the Ottawa ankle rules accurately rule out ankle fractures and can substantially reduce the need for x-rays in patients with acute ankle injuries.

To the best of our knowledge, this is the first systematic review and meta-analysis to investigate the overall diagnostic accuracy of the OAR in acute ankle injuries in adults alone. It also offers an update from previous reviews.

## Methods

The preferred reporting items for systematic review and meta-analysis protocols (PRISMA-P) were used in the development of this systematic review and meta-analysis [8].

### Search strategy and study criteria

A comprehensive systematic search of the following databases was performed on 10^th^ December 2020: SPORTdiscus via EBSCOhost, COCHRANE via Cochrane Library, MEDLINE via Ovid technologies, EMBASE via Ovid technologies, EMCARE via Ovid technologies, and SCOPUS via institutional subscription to Elsevier Science Publishers. The search inception date was set as 1992 in consideration to the publication and uptake of the OAR. Keywords were truncated as necessary, with an example search strategy provided in Supplementary file 1. Initial searches were not limited by language. The reference lists of relevant articles were then manually searched using a snowball technique to identify other potential citations.

Retrospective and prospective studies that evaluated the diagnostic accuracy of OAR when implemented by health professionals were included if they: (1) assessed and reported the psychometric properties of the OAR; (2) had an empirical research design; and (3) were peer-reviewed. Studies were excluded if participants were aged less than 18 years, the full text was not accessible, documents were unable to be accessed or translated in English, researchers were unable to distinguish between the Ottawa ankle and midfoot rules or there were insufficient data to create 2 × 2 tables on diagnostic accuracy.

### Study selection and data extraction

The study selection and data extraction processes were performed by two independent reviewers.

Search results were initially managed in EndNote (version X9, Clarivate Analytics, PA, USA). After removing duplicated articles, citations were exported into Covidence software (Veritas Health Innovation, Melbourne, Australia). Titles and abstracts were screened based on the pre-determined criteria. The remaining full texts were reviewed for eligibility and included or excluded based on the preset criteria; conflicts were resolved by consensus, or by a third reviewer if consensus could not be reached.

Data extraction included author name, publication year, region/country where study was performed, study design (retrospective or prospective), sample size, sampling technique (consecutive or convenience), site of study, patient characteristics (e.g. mean age), reference standard for the OAR, and characteristics of person who interpreted outcomes (e.g. profession).

### Critical appraisal of methodological bias

**Fig. 2:**
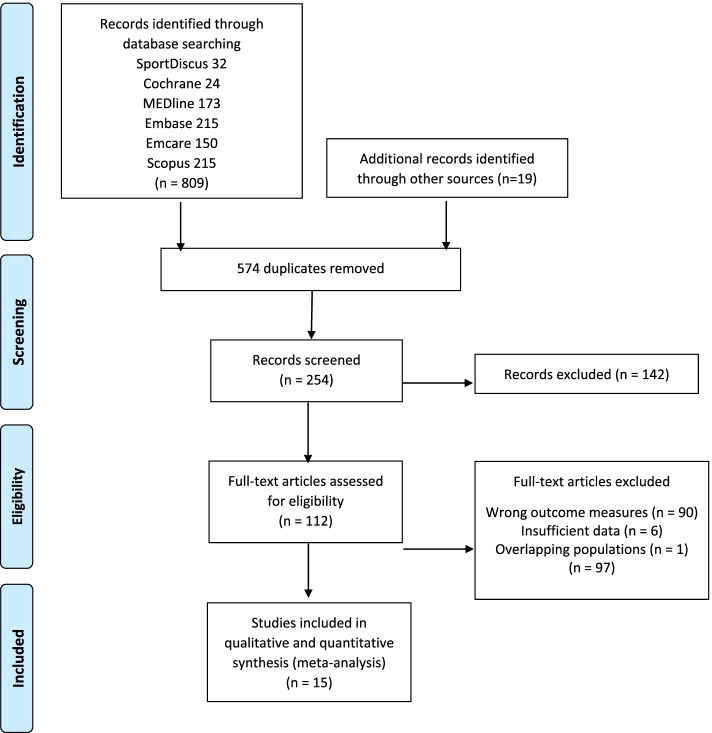
Modified PRISMA flow diagram

Study quality (i.e., risk of bias) was assessed by two reviewers independently using the revised instrument for Quality Assessment of Diagnostic Accuracy Studies 2 (QUADAS-2) tool [9]. The QUADAS-2 tool consists of seven domains assessing Risk of bias (patient selection, index test, reference standard and flow of timing) and Applicability concerns (patient selection, index test and reference standard), (Fig. 2). Each item in the QUADAS-2 tool was scored as yes, no, or unclear when there was insufficient information provided to make a precise judgement. Disagreement was resolved by consensus, or by a third reviewer if consensus could not be reached.

### Data synthesis and statistical analysis

Descriptive statistics were used to identify relevant study characteristics such as author, country, study design and participant characteristics. Analysis investigating psychometric outcomes were performed using Meta-DiSc (version 1.4 for Windows™, XI Cochrane Colloquium). For each study, the following measures of test accuracy were retrieved and computed: sensitivity, specificity, positive likelihood ratios and negative likelihood ratios, and the area under the summary receiver operating curve (SROC) (with corresponding 95% confidence interval (CI)). A SROC and area under the curve (AUC) were generated to graphically display the overall accuracy of OAR for the diagnosis of acute ankle fractures. Heterogeneity of our meta-analysis were evaluated in several ways. The Cochran Q test was used for summary estimates (with *p* < 0.05 indicating heterogeneity) and the Higgins inconsistency index (I^2^) to indicate the percentage of variance in meta-analysis [10]. Values of I^2^ equal to 25%, 50% and 75% were identified as low, moderate, and high heterogeneity respectively [11, 12]. Where heterogeneity existed, random effects modelling was used for meta-analysis. Spearman correlation coefficient investigated the threshold effect between sensitivity and false-positive rates (< 0.6 indicating considerable effect), confirmed through visual assessment of couple forest plots. Publication bias was not assessed as no accepted method exists for evaluation in a meta-analysis of diagnostic test accuracy studies [13].

## Results

A total of 254 unique articles were identified through the systematic search of the literature, with 142 removed based on inclusion/exclusion criteria and a further 97 excluded due to incorrect outcome measures reported, insufficient reporting of results for our purpose (i.e., could not construct 2 × 2 tables), and overlapping populations (where the same study participants were included across multiple studies) leaving 15 studies to be included in this review. A diagram adapted from the PRISMA statement, summarising the search and screening method, is presented in Fig. 1 [14].

### Study characteristics

The patient and study characteristics of the studies included in the meta-analysis are summarised in Table 1. Fifteen eligible studies remained for the data extraction phase, which included data for 8,560 adult participants from studies conducted in 13 countries [6, 15–28]. The average age of the enrolled population ranges from 24.9 to 51 years. Can and colleagues [18] specified two mean ages: 51 years old for those with fracture present, and 38 years old for those with fracture absent [18]. Thirteen cohort studies were included (three were retrospective in nature [6, 19, 20], nine were prospective studies [15–17, 21, 22, 24, 25, 28, 29], one reported both retrospective and prospective outcomes [27], one randomised controlled trial [16], and one non-randomised control trial [26]. The sample size ranged from 67 to 2500 participants. Fourteen studies adopted the consecutive recruitment technique whereas one study used a convenience sampling method [21]. Twelve studies had radiologists and/or ED physicians as the reference standard for the interpretation of radiographs, whereas it was unclear who did the reporting of the radiographs in four [19, 23, 24, 27] studies.

**Table 1:** Study characteristics and design

**Study**	**Year**	**Region**	**Study design**	**Sample size**	**Age (mean** ± STDEV)	**Sampling method**	**Reference standard (Radiography and/or follow up)**	**Radiograph interpretation (radiologist or ED physician)**	**Follow-up interpretation**
**Auley 1998 ** [1]	1998	France	Prospective validation survey	416	34 [range: 18–90]	Consecutive	Radiography	ED physician at time of visit + blinded radiologist (for OAR performance)	No follow up
**Beceren 2013 ** [3]	2013	Turkey	Randomized prospective	962	30.3 ± 13.2	Consecutive	Radiography	Orthopaedic surgery resident	N/R
**Broomhead 2003 ** [5]	2003	Australia	Prospective validation study	333	34.7 [range: 18.1–84.8]	Consecutive	Radiography	Radiologist	No follow up
**Can 2008 ** [6]	2008	Zurich	Prospective cohort study	251	51 ± 21 (fracture present) 38 ± 17 (fracture absent)	Consecutive	Radiography	Radiologist and emergency physicians	N/R
**Cheng 2016 ** [7]	2016	Australia	Retrospective review	404	38.5	Consecutive	Radiography	N/R	N/R
**Daş 2016 ** [8]	2016	Turkey	Retrospective case–control analysis	405	37.5	Consecutive	Radiography	Radiologist and Orthopaedic surgeon (blinded to OAR status)	No follow up
**Glas 2002 ** [12]	2002	Amsterdam	Prospective comparative study	647	35 ± 14	Consecutive	Radiography	Radiologist and trauma surgeon	N/R
**Gomes 2020 ** [13]	2000	Australia	Retrospective review	262	38 ± 13.8	Unclear	Radiography	Radiologist	N/R
**Lucchesi 1995 ** [16]	1995	Michigan	Prospective validation study	484	38 [range: 18–81]	Convenience	Radiography	Radiologist	No follow up
**Papacostas 2001 ** [21]	2001	Greece	Prospective survey	79	29 ± 9.6	Consecutive	Radiography	Radiologist	No follow up
**Rosin 1999 ** [22]	1999	South Korea	Retrospective	67	24.9 [range: 19–41]	Consecutive	Radiography	N/R	N/R
**Santelli 2008 ** [24]	2008	France	Prospective	248	31.8 ± 15.9	Consecutive	Radiography	Radiologist	N/R
**Salt 1997 ** [23]	1997	UK	Prospective	324	Above 18	Consecutive	Radiography	N/R	No follow-up
**Stiell 1994 ** [25]	1994	Canada	Non-randomized controlled trial	498	37 ± 16	Consecutive	Radiography	Radiologist	Telephoned at 10 days or asked to return for re-assessment
**Verma 1997 ** [28]	1997	US	Retrospective	2500	Above 18	Consecutive	Radiography	N/R	Medical record review
**Verma 1997 ** [28]	1997	US	Prospective	759	Above 18	Consecutive	Radiography	N/R	Telephone or medical record review
**Wang 2013 ** [23]	2013	China	Prospective	183	36.6 [range:18–70]	Consecutive	Radiography	ED physician	3D CT

*ED* Emergency department, *N/R* Not recorded, *UK* United Kingdom, *US* United States of America, *3D* Three-dimensional, *CT* Computed tomography

### Critical appraisal of methodological bias

**Fig. 3:**
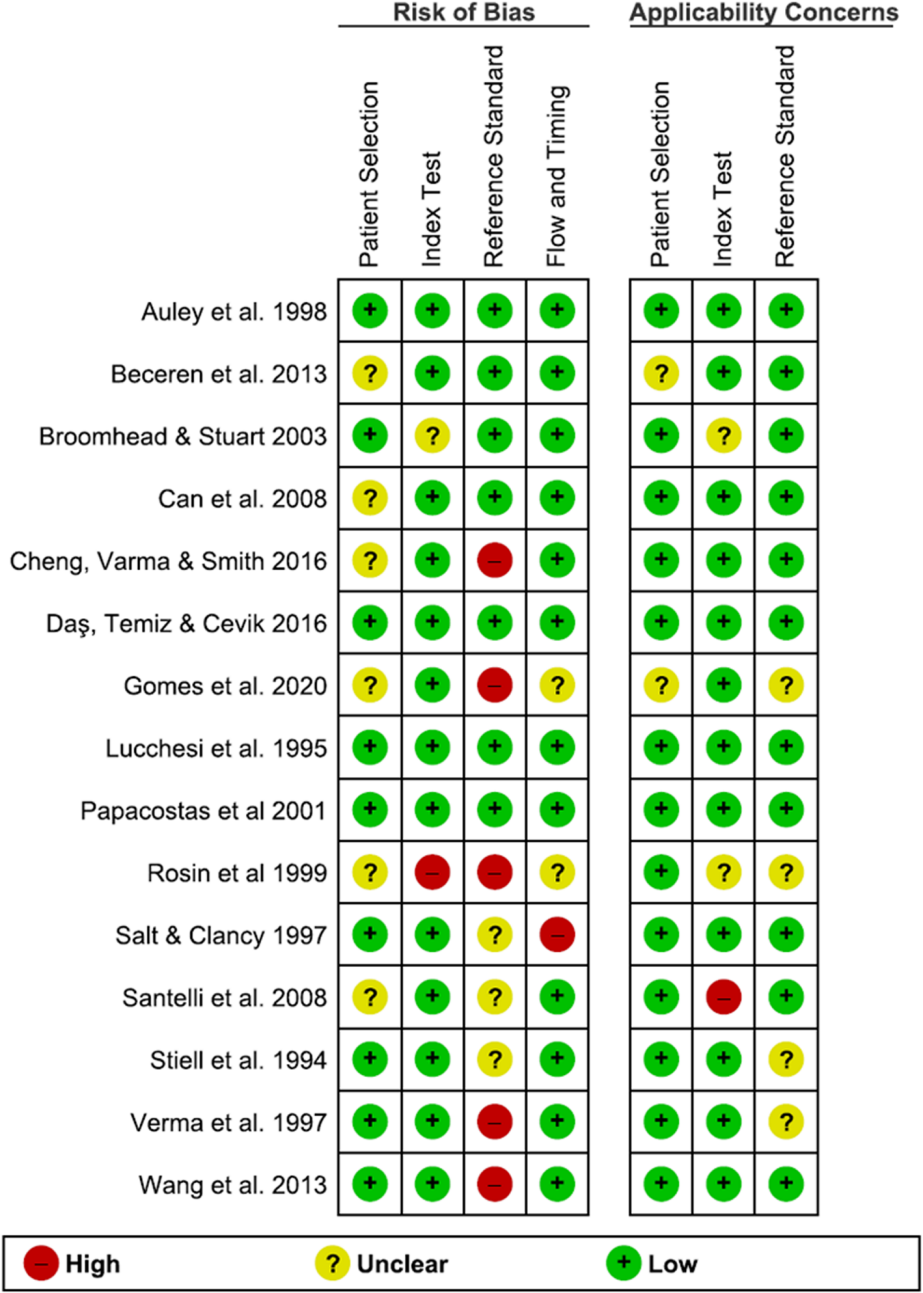
Risk of bias and applicability concerns’ summary from the QUADAS-2 tool for 15 studies included in meta-analysis

**Fig. 4:**
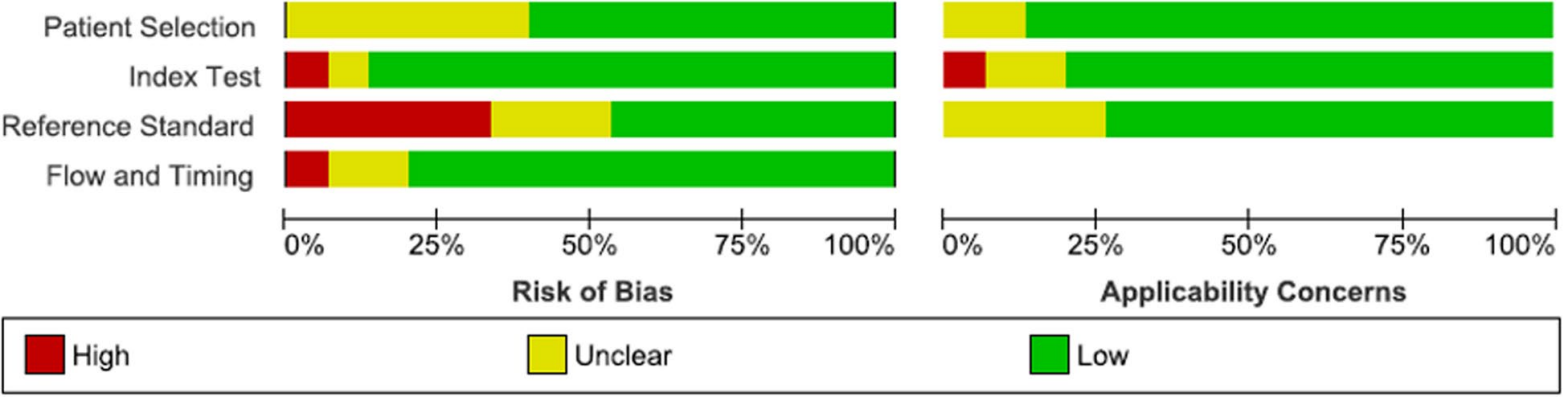
Risk of bias and applicability concerns’ graph from the QUADAS-2 tool for 15 studies included in meta-analysis

Overall, eight studies presented a low risk of bias and concern regarding applicability [15–18, 20–22, 26]. Four studies received a lower quality rating for applicability, mainly due to lack of information in the radiography interpretation (see Figs. 3 and 4) [19, 23, 27, 28]. We believe this presents an unclear risk of bias to the reference task as the radiologist interpretation remains the gold standard. In six articles, it was unclear whether the patient population was consecutively or randomly sampled [6, 16, 18, 19, 23, 25]. This presents a high risk of bias for patient selection as patients were enrolled using an unclear methodology and a non-randomized allocation to control and intervention groups might have been conducted. Blinding was not reported in eight studies which presents a high risk of bias to the results [6, 19, 23–28].

### Quantitative synthesis and assessment of heterogeneity

Data on the diagnostic accuracy of the OAR could be extracted from all 15 included studies. Data on the sensitivity and specificity reported in 15 studies are presented in Table 2. The sensitivity and specificity point estimates were found to range from 59 to 100% and 2 to 69%, respectively, and demonstrated high between-study heterogeneity (sensitivity: I^2^ = 94.3%, *p* < 0.01; specificity: I^2^ = 99.2%, *p* < 0.01). However, due to a very small number of false-negative outcomes, this should be interpreted with caution.

**Table 2:** Diagnostic accuracy results from the included studies

**Study**	**TP**	**FP**	**FN**	**TN**	**Sensitivity** **(95% CI)**	**Specificity** **(95% CI)**	**Positive likelihood ratios** **(95% CI)**	**Negative likelihood ratios** **(95% CI)**	**Diagnostic Odds Ratio** **(95% CI)**
Auley 1998 [1]	48	171	1	137	0.98 (0.89–1.00)	0.44 (0.39–0.50)	1.76 (1.58–1.97)	0.05 (0.01–0.32)	38.46 (5.24–282.17)
Beceren 2013 [3]	235	203	83	445	0.74 (0.69–0.79)	0.69 (0.65–0.72)	2.36 (2.07–2.69)	0.38 (031–0.46)	6.21 (4.60–8.38)
Broomhead 2003 [5]	43	187	0	35	1.00 (0.92–1.00)	0.16 (0.11–0.21)	1.18 (1.10–1.26)	0.07 (0.00–1.14)	16.47 (0.99–273.79)
Can 2008 [6]	33	173	0	45	1.00 (0.89–1.00)	0.21 (0.15–0.27)	1.24 (1.15–1.35)	0.07 (0.00–1.12)	17.57 (1.06–292.27)
Cheng 2016 [7]	68	263	3	45	0.96 (0.88–0.99)	0.15 (0.11–0.19)	1.12 (1.05–1.20)	0.29 (0.09–0.90)	3.88 (1.17–12.86)
Daş 2016 [8]	61	190	1	153	0.98 (0.91–1.00)	0.45 (0.39–0.50)	1.78 (1.61–1.96)	0.04 (0.01–0.25)	49.12 (6.73–358.42)
Glas 2002 [12]	44	119	30	69	0.59 (0.47–0.71)	0.37 (0.0–0.44)	0.94 (0.76–1.17)	1.10 (0.79–1.54)	0.85 (0.49–1.48)
Gomes 2020 [13]	88	278	5	51	0.95 (0.88–0.98)	0.16 (0.12–0.20)	1.12 (1.05–1.20)	0.35 (0.14–0.84)	3.23 (1.25–8.34)
Lucchesi 1995 [16]	9	49	0	21	1.00 (0.66–1.00)	0.30 (0.20–0.42)	1.36 (1.11–1.68)	0.17 (0.01–2.52)	8.25 (0.46–148.27)
Papacostas 2001 [21]	7	15	3	42	0.70 (0.35–0.93)	0.74 (0.60–0.84)	2.66 (1.47–4.82)	0.41 (0.16–1.06)	6.53 (1.49–28.57)
Rosin 1999 [22]	48	190	0	86	1.00 (0.93–1.00)	0.31 (0.26–0.37)	1.44 (1.32–1.57)	0.03 (0.00–0.52)	44.04 (2.68–722.59)
Santelli 2008 [24]	29	66	0	51	1.00 (0.88–1.00)	0.44 (0.34–0.53)	1.74 (1.48–2.06)	0.04 (0.00–0.60)	45.69 (2.73–765.63)
Salt 1997 [23]	74	244	0	247	1.00 (0.95–1.00)	0.50 (0.46–0.55)	2.00 (1.83–2.19)	0.01 (0.00–0.21)	150.83 (9.29–2447.58)
Verma 1997 (prospective) [28]	152	607	2	150	0.99 (0.95–1.00)	0.20 (0.17–0.23)	1.23 (1.18–1.28)	0.07 (0.02–0.26)	18.78 (4.60–76.65)
Verma 1997 (retrospective) [28]	270	2181	0	50	1.00 (0.99–1.00)	0.02 (0.02–0.03)	1.02 (1.01–1.03)	0.08 (0.01–1.32)	12.52 (0.77–203.57)
Wang 2013 [29]	61	65	2	55	0.97 (0.89–1.00)	0.46 (0.37–0.55)	1.79 (1.51–2.12)	0.07 (0.02–0.26)	25.81 (6.03–110.41)

*TP* True positive, *FP* False positive, *FN* False negative, *TN* True negative, *CI* Confidence interval

**Fig. 5:**
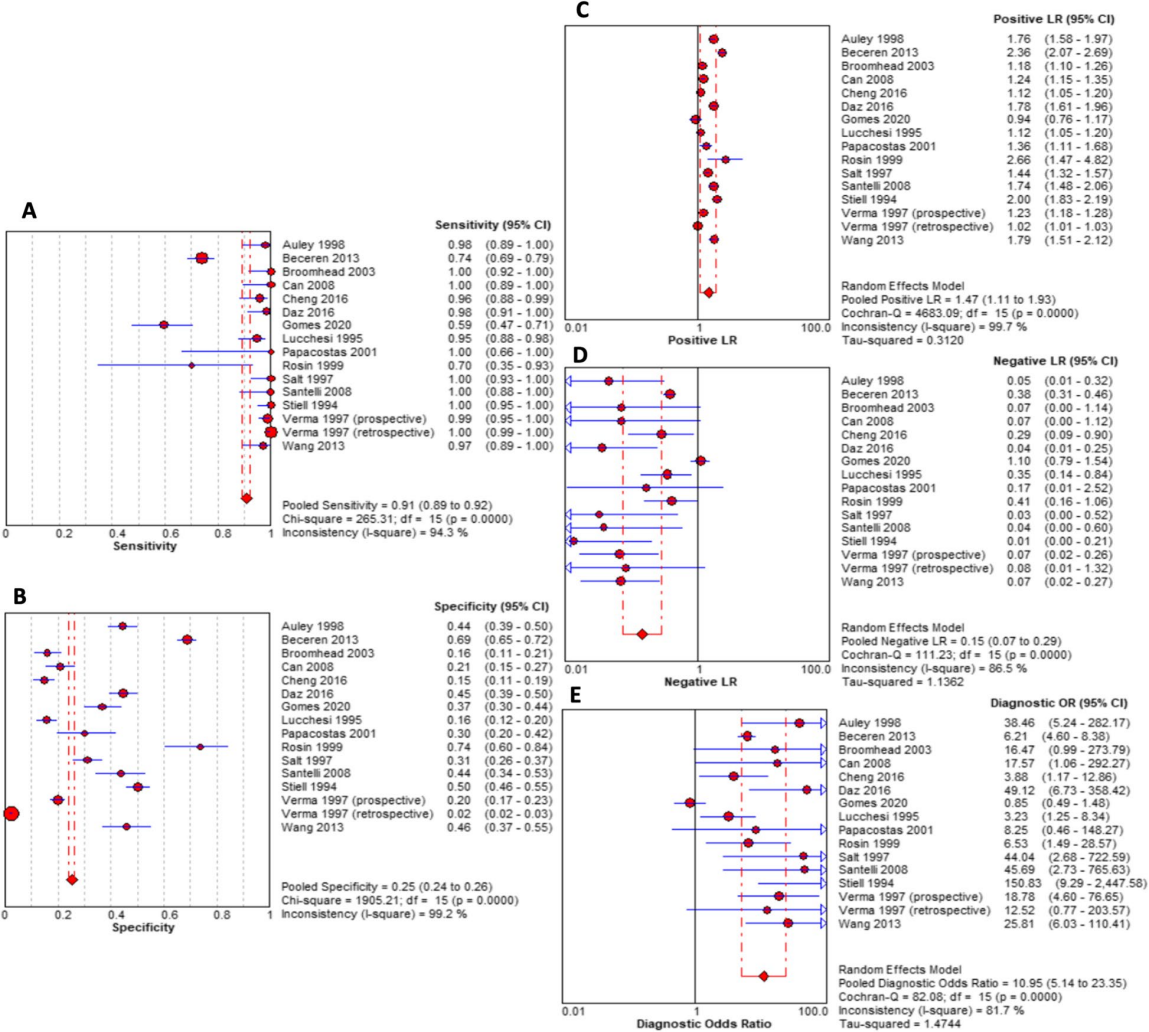
Forest plots showing pooled sensitivity (**A**), specificity (**B**), positive likelihood ratio (**C**), negative likelihood ratio (**D**) and diagnostic odds ratio (OR) (**E**). CI, confident interval; LR, likelihood ratio

**Fig. 6:**
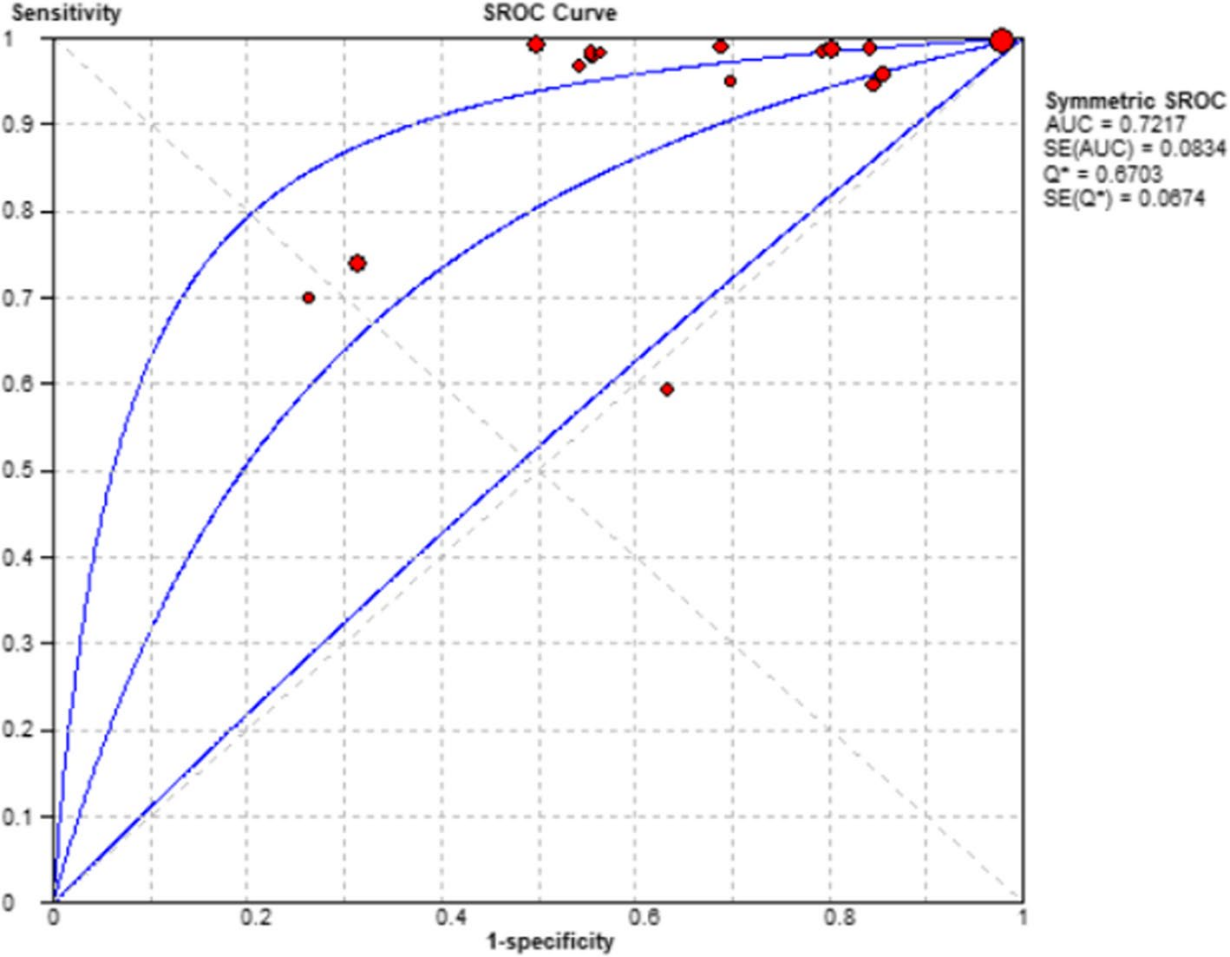
Forest plots showing diagnostic odds ratio (A) and the SROC curve (B). AUC, area under the curve; CI, confidential interval; SE, standard error; SROC, summary receiver operating characteristic curve

Due to the small number of available studies, further sub-group analyses to evaluate potential sources of heterogeneity were not performed. Using a bivariate random effects meta-analysis, pooled sensitivity of 0.91 (95% CI, 0.89 to 0.92), specificity of 0.25 (95% CI, 0.24 to 0.26), positive likelihood ratio of 1.47 (95% CI, 1.11 to 1.93), negative likelihood ratio of 0.15 (95% CI, 0.72 to 0.29) and diagnostic odds ratio of 10.95 (95% CI, 5.14 to 23.35) were calculated (see Fig. 5). Spearman correlation coefficient was 0.31, showing no evidence of a threshold effect (*p* = 0.31 [*p* > 0.05]). Figure 6 shows the SROC curve for the diagnostic value of OAR, with an AUC of 0 (Fig. 6).

## Discussion

The aim of this study was to determine the sensitivity and specificity of the OAR in diagnosing ankle fractures by means of meta-analysis of available data and provide an update to previous studies with similar purpose. The key findings are that the tool can confidently predict ankle fractures (prior to radiographic confirmation) when they exist, with high sensitivity observed (0.91 (95% CI, 0.89 to 0.92), however, with lower specificity noted (0.25 (95% CI, 0.24 to 0.26) a percentage of people without ankle fractures will be falsely predicted as having one when using these rules. Calculated positive likelihood ratios indicate that a positive finding using the OAR increases the odds of having an ankle fracture 1.5 times. The calculated negative likelihood ratio indicates that a negative finding rules out the odds of having an ankle fracture by 1.47 times These outcomes add to a growing body of knowledge regarding the OAR’s psychometric properties, specifically for the adult population.

These findings are consistent with previous SR and meta-analyses identified. In 2013, Bachmann et al. [7] found sensitivities of 99.6% (95% confidence interval 98.2% to 100.0%) (if applied within 48 h) and a calculated specificity range from 47.9% (interquartile range 42.3% to 77.1%) in studies with a prevalence of fracture below the 25th percentile of all studies, to 26.3% (19.4% to 34.3%) in studies of combined assessment. Jonckheer et al. [30] found sensitivity and specificity values of 92% to 100% and from 16 to 51%, respectively. Beckencamp et al. [5] found sensitivity and specificity values of 99.4% (97.9% to 99.8%) and 32.3% (28.8% to 42.3%) respectively. Interestingly, these values were not significantly improved with an analysis of only those papers evaluated as being of low risk of bias (96.4% (83.7% to 99.3%) and 31.9% (8.3% to 70.7%)). Our decision to not undertake a sub-analysis based on quality was due to this finding.

The calculated sensitivity for our study is lower (with a broader value range) than those reported by Bachmann et al. [7] and Beckencamp et al. [5] above. Whilst both these studies included children in their sample, this is unlikely to account for differences between their findings and ours given a sub-analysis by Beckencamp et al. [5] found that the adult-only sensitivity was superior to the children-only sample. The lower findings calculated in this study does appear driven by the inclusion of three papers [6, 16, 23]. There may be a number of reasons that explain their effect. The study by Rosin et al. [23] study included a military only population. This may have affected the clinical decision making (i.e., need to rule out fracture to continue military exercises/training, improved access to radiographic services, no cost to patient, patient’s reporting of symptoms etc.). It appears that Beceren et al. [16], in their study comparing the OARs with the Bernese Ankle Rules, used modified criteria which included palpation of the Navicular and the Metatarsal. Subsequently, positive fractures of these areas were also included, with them reporting Metatarsal fractures to be the most commonly found. Gomes et al. [6] was a retrospective audit of referrals, and thus may have lacked sufficient detail regarding reporting of symptoms or other indicators, as well as a bias, with those not referred for x-ray not being included in the study. Further to this, all three of these studies displayed some concern regarding patient selection section on the quality assessment tool.

Similar differences were seen in the lower calculated specificity values within this study, with this value primarily driven by a retrospective study undertaken by Verma et al. [27]. The values reported in studies by Rosin et al. [23] and Beceren et al. [16] were also quite distinct from other studies, supporting the notion of different participant selection could have contributed. It is not uncommon for diagnostic criteria of high sensitivity to then display lower levels of specificity. It is important to note that OAR are largely applied in an acute setting, within 48 h of injury. It is then questionable whether the overcautious nature of the criteria is relevant given that management is unlikely to be much different, particularly in cases without high levels of pain.

Negative OARs results tended to rule out the need to obtain radiographs. One of the weaknesses of the Ottawa ankle rues is the low specificity of the test, which leads to many false positive clinical findings. This suggests that a positive OAR result cannot be the sole indicator for obtaining radiographs. In line with an evidence-based approach to practice, the OAR should be used in conjunction with clinical reasoning and judgment. When the OAR result is negative, the emergency clinician can be confident that a fracture is not present. However, when the result is positive, they must consider the possibility of a false positive result. The main reason for utilizing this diagnostic tool in the clinical setting is to eliminate unnecessary radiographs. The meta-analysis suggests that a negative OAR result is a a good predictor in ruling out a fracture but is not clinically reliable in doing so and must be used in conjunction with judicious clinical reasoning. The OARs still hold the highest sensitivity (up to 100% in some studies [5, 7]) compared to the Bernese Ankle Rules (sensitivity of 94%) and Leiden ankle rule (80%) [29].

We believe our literature search, study selection and quality assessment of the included studies were comprehensive and reliable. There are some limitations with our systematic review and meta-analysis. Our study population focuses on adult patients (above 18 years of age), studies with patients under 18 years of age were excluded. Therefore, subgroup analyses of the paediatric population were not possible. Moreover, some studies also included patients over the age of 80 [1, 5, 16]; this might have caused variability to the results of OAR as elderly patients might have a reduced sensitivity to pain [31]. Also, our search strategy excluded non-English articles and conference abstracts, which could have influenced the results slightly. Thirdly, a potential source of heterogeneity is the threshold effect (i.e., the relationship between sensitivity and specificity across studies) in meta-analyses. The spearman correlation coefficient in our analysis confirmed that there is no threshold effect related to heterogeneity; however, we reported a heterogenous effect across different studies with a Chi-square ranging from 81.7% to 99.7%. This is primarily due to the low number of false-negative results across all included studies. Lastly, one study [3] had an orthopaedic surgery resident interpreting the radiographs. This may have introduced some bias to the overall results due to a potential Hawthorne effect. Based on our risk of bias assessment using the QUADAS-2 tool, most studies rated quite well, with the largest issues of concern being uncertainty regarding participant selection (largely whether participants were enrolled consecutively or not) and issues with the reference standard (articles did not specify the x-ray criteria). With a few studies there was uncertainty regarding blinding of the person applying the reference standard.

Since their introduction by Stiell et al. [4] in 1992, the OAR have been utilised worldwide now for almost 30 years. They comprise a core component of guidelines incorporated in many countries. Consequently, the use of the OAR rules has been researched across many sites in many countries. Despite this, only 15 papers met our criteria and subsequently underwent quality review and meta-analysis. This is surprising given its utilisation worldwide and the proliferation of research generally. This may be reflective of a much lower uptake than anticipated or a publication bias or other, unknown reasons. It does suggest, however, that further high-quality research in ongoing use of this tool is warranted.

## Conclusion

A systematic review of the literature and meta-analysis of results determined that the application of the OAR tool in an adult population was observed to have high sensitivity and could be used confidently to rule out ankle fractures and reduce the need for unnecessary radiographs. The specificity rate was lower, increasing the likelihood of false-positive outcomes (i.e., resulting in the prediction of an ankle fracture that does not exist). A positive finding using the OAR increases the odds of having an ankle fracture 1.5 times. Low specificity rates indicate false positive results, which suggests that while a negative OAR result is a relatively good predictor in ruling out a fracture, it is not clinically reliable in ruling out a fracture. These findings add to a growing body of knowledge that supports the use of the OAR as a cost-effective tool to reduce unnecessary radiographic referral, when used in conjunction with the emergency clinician’s clinical reasoning and judgement. Implementation and uptake of the tool will improve efficiency, lower medical costs and reduce waiting times for those attending health services following ankle trauma.

## Supplementary Information

**Additional file 1. **Search strategy.

## Data Availability

The datasets used and/or analysed during the current study are available from the corresponding author on reasonable request.

## Abbreviations

AUC: Area under the curve
CI: Confidence interval
ED: Emergency Department
FN: False negative
FP: False positive
I^2^: Higgins inconsistency index
LR: Likelihood ratio
MeSH: Medical subject headings
N/R: Not Recorded
OAR: Ottawa Ankle Rules
PRISMA-P: Ethics approval Preferred reporting items for systematic review and meta-analysis protocols
QUADAS-2: Quality Assessment of Diagnostic Accuracy Studies 2
SROC: Summary receiver operating curve
TP: True positive
TN: True negative
