# Supplementary material for: Diagnostic accuracy of the Ottawa ankle rule to exclude fractures in acute ankle injuries in adults: a systematic review and meta-analysis
Source: BMC Musculoskelet Disord. 2022 Sep 23;23:885. doi: 10.1186/s12891-022-05831-7 (PMC9502997; doi:10.1186/s12891-022-05831-7)
Supplement: Supplementary file 1 — Additional file 1. Search strategy. [file 12891_2022_5831_MOESM1_ESM.docx]

**Supplementary File 1:**

Search strategy

| Database | Search terms | Number of hits |
| --- | --- | --- |
| SPORTDiscus via EBSCO Host | All fields: "ottawa ankle rule*" OR "ottowa ankle rule*" OR (ottawa N4 ankle*) OR (ottowa N4 ankle*) | 32 |
| Cochrane via Cochrane Library | All fields: ((ott?wa NEXT ankle NEXT rule*) OR (ott?wa NEAR/4 ankle*)) | 24 |
| MEDLINE via Ovid technologies | All fields: (ott#wa ankle rule* or (ott#wa adj4 ankle*)) | 173 |
| EMBASE via Ovid technologies | All fields: (ott#wa ankle rule* or (ott#wa adj4 ankle*)) | 215 |
| EMCARE via Ovid technologies | All fields: (ott#wa ankle rule* or (ott#wa adj4 ankle*)) | 250 |
| Scopus | All fields: (ott#wa ankle rule* or (ott#wa adj4 ankle*)) | 215 |
